# Clinical significance and risk factors for new onset and recurring atrial fibrillation following cardiac surgery - a retrospective data analysis

**DOI:** 10.1186/s12871-017-0455-7

**Published:** 2017-12-02

**Authors:** Hristo Todorov, Inka Janssen, Stefanie Honndorf, Daniela Bause, Antje Gottschalk, Silke Baasner, Thomas Volkert, Valentin Faerber, John F. Stover, Martin Westphal, Björn Ellger

**Affiliations:** 1 Institute for Molecular Bioinformatics, Johann Wolfgang Goethe-University Frankfurt, Robert-Mayer-Str. 11-15, 60325 Frankfurt am Main, Germany; 2 Fresenius Kabi Deutschland GmbH, Else-Kröner-Str. 1, 61352, Bad Homburg, Germany; 3 Department of Anesthesiology, Intensive Care and Pain Medicine, University Hospital Muenster, Albert-Schweitzer-Campus 1, Building A1, 48149 Muenster, Germany; 4 Department of Anesthesiology, Intensive Care and Pain Medicine, Klinikum Westfalen, Am Knappschaftskrankenhaus 1, 44309 Dortmund, Germany

**Keywords:** Postoperative atrial fibrillation, CABG, Valve surgery, Inflammation, C-reactive protein, Positive fluid balance, Phosphate

## Abstract

**Background:**

Although mortality after cardiac surgery has significantly decreased in the last decade, patients still experience clinically relevant postoperative complications. Among others, atrial fibrillation (AF) is a common consequence of cardiac surgery, which is associated with prolonged hospitalization and increased mortality.

**Methods:**

We retrospectively analyzed data from patients who underwent coronary artery bypass grafting, valve surgery or a combination of both at the University Hospital Muenster between April 2014 and July 2015. We evaluated the incidence of new onset and intermittent/permanent AF (patients with pre- and postoperative AF). Furthermore, we investigated the impact of postoperative AF on clinical outcomes and evaluated potential risk factors.

**Results:**

In total, 999 patients were included in the analysis. New onset AF occurred in 24.9% of the patients and the incidence of intermittent/permanent AF was 59.5%. Both types of postoperative AF were associated with prolonged ICU length of stay (median increase approx. 2 days) and duration of mechanical ventilation (median increase 1 h). Additionally, new onset AF patients had a higher rate of dialysis and hospital mortality and more positive fluid balance on the day of surgery and postoperative days 1 and 2. In a multiple logistic regression model, advanced age (odds ratio (OR) = 1.448 per decade increase, *p* < 0.0001), a combination of CABG and valve surgery (OR = 1.711, *p* = 0.047), higher C-reactive protein (OR = 1.06 per unit increase, *p* < 0.0001) and creatinine plasma concentration (OR = 1.287 per unit increase, *p* = 0.032) significantly predicted new onset AF. Higher Horowitz index values were associated with a reduced risk (OR = 0.996 per unit increase, *p* = 0.012). In a separate model, higher plasma creatinine concentration (OR = 2.125 per unit increase, *p* = 0.022) was a significant risk factor for intermittent/permanent AF whereas higher plasma phosphate concentration (OR = 0.522 per unit increase, *p* = 0.003) indicated reduced occurrence of this arrhythmia.

**Conclusions:**

New onset and intermittent/permanent AF are associated with adverse clinical outcomes of elective cardiac surgery patients. Different risk factors implicated in postoperative AF suggest different mechanisms might be involved in its pathogenesis. Customized clinical management protocols seem to be warranted for a higher success rate of prevention and treatment of postoperative AF.

**Electronic supplementary material:**

The online version of this article (10.1186/s12871-017-0455-7) contains supplementary material, which is available to authorized users.

## Background

Patients undergoing cardiac surgery usually have various accompanying risk factors for adverse outcome including hypertension, chronic congestive heart failure, myocardial infarction and previous cardiac surgery [1]. Due to improved patient care over the last decade risk-adjusted mortality for the most common type of cardiac surgery, coronary artery bypass grafting (CABG), has significantly decreased [2]. Consequently, the focus of research has shifted to the prevention of other peri- and postoperative complications with the aim to further improve clinical outcomes of these patients.

One complication following cardiac surgery, which is increasingly gaining interest in clinical research, is atrial fibrillation (AF). The reported incidence following CABG ranges from 15% to approximately 48% [3–12]. The incidence after valve reconstruction or replacement surgery varies between 18% to approximately 74% [4, 6, 13–17]. Following a combined procedure of myocardial revascularisation and valve surgery, postoperative AF incidence is reported in the range of 18% to 81% [4, 6, 9, 14, 18].

Increasing age is the most consistent risk factor [3–5, 7, 8, 11–14, 18–20] with every additional decade adding about 50% to the risk of developing AF [4, 18, 20]. Further common pre-operative risk factors include decreased left-ventricular ejection fraction [8, 15], left atrial enlargement [4, 7], chronic obstructive pulmonary disease [8, 11, 13, 18, 20] and peripheral vascular disease [5, 11, 13, 20].

Postoperative AF is associated with increased intensive care unit (ICU) and hospital length of stay [3, 8, 9, 11, 13, 14, 18, 19], an increased incidence of stroke following surgery [11, 13, 15, 18], higher rates of multiple organ failure [14], renal failure [10], readmission to the ICU [18, 19], pneumonia [14] and ventricular arrhythmias [13]. This association peaks in increased in-hospital mortality [11] and reduced long-term survival [10, 11, 16].

Despite intensive research, the pathogenesis of postoperative AF is still not fully understood. Whereas previous research has primarily focused only on new onset AF, the impact of a recurrent postoperative episode on clinical outcome in patients with pre-existing AF and the accompanying risk factors deserve further investigation.

We hypothesized that postoperative AF occurs along with inflammation and that positive fluid balance, typically seen in cardiac surgery patients, might also be a contributing factor for developing postoperative AF.

In this retrospective study, we analyzed the occurrence and clinical significance of AF in a large sample of 1000 patients following elective CABG, valve surgery or a combination of both procedures. The aim was to elucidate if patients with new onset AF and patients with known preoperative AF history who had a recurrent episode following surgery (intermittent/permanent AF) have significantly different clinical outcome and if distinct risk factors are associated with both types of postoperative arrhythmia.

## Methods

The present study was designed as a retrospective analysis of patients who underwent either on-pump CABG, valve surgery or a combination of on-pump CABG and valve surgery at the University Hospital of Muenster in the period between April 2014 and July 2015. The collective term valve surgery refers to either reconstruction or replacement to one of the four heart valves or a combination of these interventions. The study was approved by the ethics committee of the Medical Association Westfalen-Lippe and the University of Muenster (Approval No. 2015–487-f-S) The need for informed consent was waived due to the non-interventional, retrospective design of the study. A screening of patient data stored at the University Hospital Muenster internal database was initiated starting from July 2015 and went backwards until 1000 patients were recruited for the analysis to ensure a sufficiently large sample. Patients were excluded based on the following criteria: Need for extracorporeal life support, emergency intervention, surgical revision, minors and pregnant women.

### Primary endpoint

The primary endpoint of the analysis was the incidence of postoperative AF and the identification of risk factors. Information about the occurrence of AF was extracted from the database using a computer algorithm or by manual inspection of patient files.

### Secondary variables

The data set included demographic information such as age, gender and weight at admission. Clinical outcome characteristics such as need for dialysis, duration of mechanical ventilation, the occurrence of postoperative atrio-ventricular (AV) block III or ventricular arrhythmias (ventricular flutter, fibrillation or tachycardia), ICU length of stay, epinephrine or dobutamine administration in the ICU and hospital mortality were also investigated. Postoperative daily values for fluid balance, Horowitz index (PaO_2_/FiO_2_ ratio) [21] as well as the clinical chemistry variables C-reactive protein (CRP), leukocyte count, total bilirubin, alanine transaminase (ALT), aspartate transaminase (AST), creatinine, phosphate, bicarbonate, sodium, potassium, chloride and calcium were extracted for each patient (if available) for up to 7 days of ICU stay post-surgery.

### Statistical analysis

Statistical analyses were performed with the statistical language R version 3.2.2 (2015, R Foundation for Statistical Computing, Vienna, Austria). Patients were allocated to four groups based on the criteria whether they had pre- or postoperative AF. Patients without any AF were assigned to the *no AF* group. The *new onset AF* group consisted of patients without pre- but with postoperative AF. Patients with pre- and without postoperative AF were allocated to the *disappeared AF* group. Lastly, patients with pre- and postoperative AF constituted the *intermittent/permanent AF* group.

Continuous variables were represented as median together with the first and third quartile. Differences between groups were evaluated with the Wilcoxon-Mann-Whitney test. For categorical variables the number of subjects in each category as well as the corresponding percentage were reported. Differences between groups were assessed using Chi-square test for independence. In case that the frequency of an observation was less than 5 in the contingency table, Fisher’s exact test was used instead.

Changes of fluid balance, leukocyte count and C-reactive protein over the course of ICU stay were graphically displayed with boxplots. Differences between groups at each day were evaluated with the help of the Wilcoxon-Mann-Whitney test.

Binary logistic regression was performed to identify risk factors for developing the two types of postoperative AF defined in the analysis (new onset AF and intermittent/permanent AF). All demographic and clinical characteristics investigated as potential predictors for postoperative AF are listed in Additional file 1. If more than one value per patient was available for a single parameter (e.g. clinical chemistry parameters), the maximal recorded value was used for model fitting. Only in the case of the Horowitz index, the minimal value for a patient was used. Multiple imputations were performed to deal with missing data. Ten sets of multiply imputed data were generated and the pooled results are reported in the final model. Candidate variables were first analyzed in univariate models. If the *p*-value was less than 0.1, the respective variable was included in a multivariable logistic regression model.

All *p*-values are two-tailed, a *p*-value <0.05 was considered as statistically significant. Due to the explorative nature of the analysis, no adjustment for multiple comparisons was performed.

## Results

### Overview of study population and patient subgroups

**Fig. 1:**
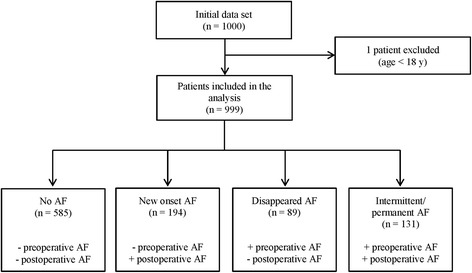
Overview of study population and subgroups for analysis. After excluding one patient who was a minor at the time of surgery, 999 patients were analysed in total. Subjects were allocated to 4 groups based on the pre- and postoperative occurrence of atrial fibrillation (AF)

After reviewing the correctness of the data, one patient <18 years old at the time of surgery was excluded from the analysis. Thus, 999 patients were evaluated in total (Fig. 1).

### Incidence of postoperative new onset and intermittent/permanent AF

Overall, 24.9% of all patients, who did not have AF prior to surgery, had postoperative new onset AF (194 out of 779 patients). After a combined procedure of CABG and valve surgery the highest rate of postoperative new onset AF was observed at 43.2% (35 out of 81 patients). Following valve surgery the incidence was 23.2% (51 out of 220 patients). After isolated CABG 22.6% of the patients developed postoperative new onset AF (108 out of 478 patients).

Overall, 131 of the 220 patients with preoperative AF had a postoperative arrhythmic episode, corresponding to an incidence of intermittent/permanent AF of 59.5%. The incidence after combined procedure was 65.5% (23 out of 35 patients). The rate of occurrence after isolated CABG was 60.7% (54 out of 89 patients). Following valve surgery 54 out of 96 patients exhibited intermittent/permanent AF, at an incidence of 56.3%.

### Patient characteristics and clinical outcome

Patients with new onset AF were significantly older compared to patients without AF (median 74 years vs 67 years, *p* < 0.001). No differences in gender and weight at admission were observed (Table 1). However, new onset AF was clearly associated with worse clinical outcomes as indicated by significantly increased ICU length of stay, duration of mechanical ventilation, incidence of dialysis and hospital mortality in patients with newly occurring postoperative arrhythmia. Furthermore, these patients were more likely to suffer from ventricular arrhythmia, to receive epinephrine in the ICU and to have undergone a combined procedure of CABG and valve surgery when compared to patients without AF (Table 1).

**Table 1:** Patient characteristics and clinical outcome of no AF vs new onset AF patients

Variable	No AF (*n* = 585)	New onset AF (*n* = 194)	*p*-Value
Age^+^	67 (58–75)	74 (66–78)	<0.001^#^
Gender [number (%)]			ns^§^
male	430 (73.5)	139 (71.6)	
female	155 (26.5)	55 (28.4)	
Weight at admission [kg]^+^	82 (72–94)	82 (73.2–91)	ns^#^
ICU length of stay [d]^+^	1.8 (1.0–2.9)	3.9 (2.1–5.9)	<0.001^#^
Duration of mechanical ventilation [h]^+^	6 (4–8)	7 (5–10)	<0.001^#^
Dialysis [number (%)]	11 (1.9)	16 (8.2)	<0.001^§^
Type of surgery [number (%)]			<0.001^§^
CABG	370 (63.2)	108 (55.7)	
Valve	169 (28.9)	51 (26.3)	
CABG + valve	46 (7.9)	35 (18.0)	
Ventricular arrhythmias [number (%)]	4 (0.7)	6 (3.1)	<0.001^$^
AV block III [number (%)]	30 (5.1)	12 (6.2)	ns^§^
Epinephrine administration [number (%)]	65 (11.1)	35 (18.0)	<0.05^§^
Dobutamine administration [number (%)]	66 (11.3)	29 (14.9)	ns^§^
Mortality [number (%)]	8 (1.4)	8 (4.1)	<0.05^$^

Data are presented as absolute number and % (in brackets) or median together with the 1st and 3rd quartile (in brackets). *P*-values over the significance threshold of 0.05 are reported as not significant (ns**)**

+ Median (1st quartile - 3rd quartile), # Wilcoxon-Mann-Whitney test, § Chi-square test for independence, $ Fisher’s exact test

*AV* Atrio-ventricular, *CABG* Coronary artery bypass grafting, *ICU* Intensive care unit

Demographics did not differ between patients with intermittent/permeant AF compared to patients with disappeared AF (Table 2). However, intermittent/permanent AF also adversely affected clinical outcomes as reflected by significantly increased ICU length of stay and duration of mechanical ventilation in this patient population (Table 2).

**Table 2:** Patient characteristics and clinical outcome of disappeared AF vs intermittent/permanent AF patients

Variable	Disappeared AF (*n* = 89)	Intermittent/permanent AF (*n* = 131)	*p*-Value
Age [years]^+^	75 (68–79)	75 (70–78)	ns^#^
Gender [number (%)]			ns^§^
male	61 (68.5)	90 (68.7)	
female	28 (31.5)	41 (31.3)	
Weight at admission [kg]^+^	80 (70–93)	82 (72–97)	ns^#^
ICU length of stay [d]^+^	2.0 (1.0–3.9)	3.9 (2.7–6.9)	<0.001^#^
Duration of mechanical ventilation [h]^+^	6 (4–9)	7 (5–11)	<0.05^#^
Dialysis [number (%)]	4 (4.5)	8 (6.1)	ns^$^
Type of surgery [number (%)]			ns^$^
CABG	35 (39.3)	54 (41.2)	
Valve	42 (47.2)	54 (41.2)	
CABG + valve	12 (13.5)	23 (17.6)	
Ventricular arrhythmias [number (%)]	0 (0)	6 (4.6)	ns^$^
AV block III [number (%)]	17 (19.1)	14 (10.7)	ns^§^
Epinephrine administration [number (%)]	19 (21.3)	43 (32.8)	ns^§^
Dobutamine administration [number (%)]	17 (19.1)	22 (16.8)	ns^§^
Mortality [number (%)]	2 (2.2)	6 (4.6)	ns^$^

Data are presented as absolute numbers and % (in brackets) or median together with the 1st and 3rd quartile (in brackets). *P*-values over the significance threshold of 0.05 are reported as not significant (ns)

+ Median (1st quartile - 3rd quartile); # Wilcoxon-Mann-Whitney test; § Chi-square test for independence $ Fisher’s exact test

*AV* Atrio-ventricular; *CABG* Coronary artery bypass grafting; *ICU* Intensive care unit

### Fluid balance and inflammation

**Fig. 2:**
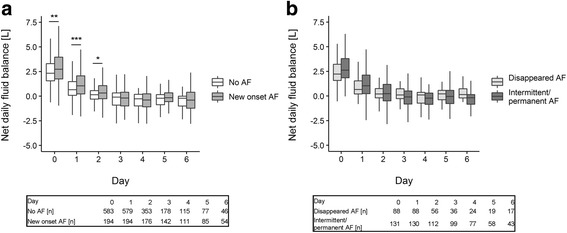
Fluid balance changes over the time course of ICU stay. **a** Patients without atrial fibrillation (AF) versus patients with new onset AF. **b** Patients with disappeared AF versus patients with intermittent/permanent AF. Data are shown as median with 1st and 3rd quartile and minimum and maximum without outliers. Number of patients on each day is displayed under the graphs. *** *p* < 0.001, ** *p* < 0.01, * *p* < 0.05 (Wilcoxon-Mann-Whitney test)

Fluid balance was the highest on the day of surgery and postoperative day 1 and continuously decreased over the course of ICU stay (Fig. 2). Patients with new onset AF were characterized by significantly higher values as compared to patients without AF on day 0 (median 2.8 L vs 2.3 L, *p* < 0.01), day 1 (median 1.04 L vs 0.66 L, *p* < 0.001) and day 2 (median 0.33 L vs 0.14 L, *p* < 0.05). A similar trend was observed in the intermittent/permanent AF group compared to the disappeared AF group (*p* > 0.062).

**Fig. 3:**
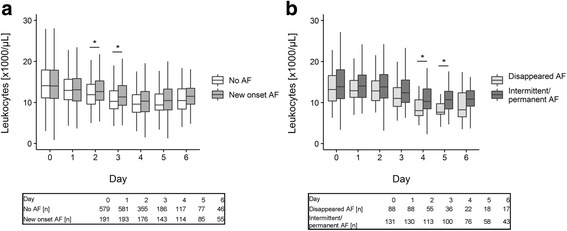
Leukocyte concentration changes over the time course of ICU stay. **a** Patients without atrial fibrillation (AF) versus patients with new onset AF. **b** Patients with disappeared AF versus patients with intermittent/permanent AF. Data are shown as median with 1st and 3rd quartile and minimum and maximum without outliers. Number of patients on each day is displayed under the graphs. * *p* < 0.05 (Wilcoxon-Mann-Whitney test)

White blood cell count was highest on the day of surgery and tended to decrease thereafter (Fig. 3). Patients with new onset AF had significantly increased leukocyte concentration in comparison to the no AF group on day 2 (median 12.65 × 10^3^/μL vs 11.89 × 10^3^/μL, *p* < 0.05) and day 3 (median 11.34 × 10^3^/μL vs 10.27 × 10^3^/μL, *p* < 0.05). In addition, patients in the intermittent/permanent AF group had a significantly increased white blood cell count relative to the disappeared AF group on day 4 (median 10.29 × 10^3^/μL vs 8.05 × 10^3^/μL, *p* < 0.05) and day 5 (median 10.69 × 10^3^/μL vs 7.65 × 10^3^/μL, *p* < 0.05).

**Fig. 4:**
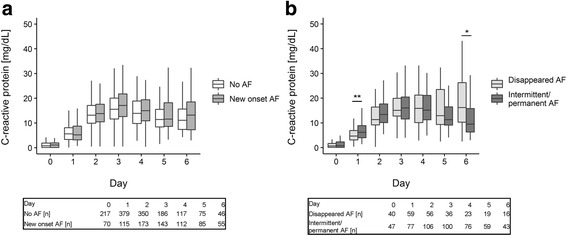
C-reactive protein concentration changes over the course of ICU stay. **a** Patients without atrial fibrillation (AF) versus patients with new onset AF. **b** Patients with disappeared AF versus patients with intermittent/permanent AF. Data are shown as median with 1st and 3rd quartile and minimum and maximum without outliers. Number of patients per day is displayed under the graphs. ** *p* < 0.01, * *p* < 0.05 (Wilcoxon-Mann-Whitney test)

Plasma concentrations of CRP continuously increased and peaked on postoperative day 3 in all groups (Fig. 4). A trend towards higher CRP levels was observed in patients with new onset AF compared to subjects without this arrhythmia (*p* > 0.10). CRP was significantly increased in the intermittent/permanent AF group when compared to the disappeared AF group on day 1 (median 6.2 mg/dL vs 4.7 mg/dL, *p* < 0.01). In contrast, patients with disappeared AF had significantly higher CRP concentration versus the intermittent/permanent AF group on day 6 (median 16.2 mg/dL vs 9.5 mg/dL, *p* < 0.05).

### Risk factors for postoperative AF

Multiple logistic regression analysis identified significant risk factors for new onset AF, even after adjusting for the most powerful predictor, age (OR = 1.448 per decade, *p* < 0.0001, Table 3). Whereas every unit rise in CRP increased the risk for new onset AF by about 6%, a unit increase in creatinine levels increased the odds by about 29%. A combination of CABG and valve surgery (OR = 1.711, *p* = 0.047) also represented a significant risk factor relative to CABG alone. Conversely, higher Horowitz index values (OR = 0.996 per unit increase, *p* = 0.012) were significantly associated with reduced occurrence of postoperative new onset AF. Univariate comparisons between the no AF and new onset AF groups for the significant predictors plasma creatinine concentration and Horowitz index on different postoperative days can be seen in Additional file 2: Figure S1 and Figure S2.

**Table 3:** Multiple logistic regression analysis for postoperative new onset AF as outcome variable

Variable	OR (95% CI)	*p*-Value
Age (per decade)	1.448 (1.241–1.76)	<0.0001
Type surgery (relative to CABG)		
Valve	1.396 (0.924–2.107)	0.113
CABG + valve	1.711 (1.008–2.903)	0.047
Ventricular arrhythmias	3.55 (0.859–14.66)	0.08
Epinephrine administration	1.121 (0.662–1.9)	0.67
ALT concentration	1.0 (0.999–1.0004)	0.856
C-reactive protein concentration	1.06 (1.033–1.088)	<0.0001
Creatinine concentration	1.287 (1.022–1.621)	0.032
Calcium concentration	1.01 (0.977–1.045)	0.547
Fluid balance (per litre)	1.078 (0.962–1.207)	0.195
Horowitz index	0.996 (0.993–0.999)	0.012

Data are shown as odds ratios (OR) together with the 95% confidence interval (CI) and the corresponding p-value. For ALT, C-reactive protein, creatinine, calcium concentrations and fluid balance, the maximal recorded value per patient was used for model fitting. For the Horowitz index, the minimal recorded value was used for model estimation. Results from the univariate analyses are shown in Additional file 1: Table S1

*AF* Atrial fibrillation; *ALT* Alanine transaminase, *CABG* Coronary artery bypass grafting

In a separate multiple logistic regression model, higher creatinine concentration was significantly associated with increased risk for intermittent/permanent AF (OR 2.125 per unit increase, *p* = 0.022, Table 4). Interestingly, every unit increase in phosphate concentration reduced the odds for intermittent/permanent by approximately 48% (OR = 0.522 per unit increase, *p* = 0.003). Univariate comparisons between patients with disappeared AF and intermittent/permanent AF on different postoperative days for the two significant predictors can be seen in Additional file 2: Figure S1 and Figure S3.

**Table 4:** Multiple logistic regression analysis for intermittent/permanent AF as outcome variable

Variable	OR (95% CI)	*p*-Value
AV block III	0.511 (0.211–1.235)	0.135
Epinephrine administration	1.03 (0.456–2.33)	0.942
C-reactive protein concentration	1.032 (0.984–1.084)	0.192
Leukocytes concentration	0.999 (0.94–1.061)	0.976
Creatinine concentration	2.125 (1.114–4.052)	0.022
Fluid balance	1.2 (0.942–1.527)	0.139
Phosphate concentration	0.522 (0.342–0.795)	0.003
Sodium concentration	1.035 (0.92–1.163)	0.565
Chloride concentration	1.049 (0.939–1.17)	0.396
Horowitz index	0.996 (0.99–1.001)	0.12

Data are shown as odds ratios (OR) together with the 95% confidence interval (CI) and the corresponding *p*-value. For C-reactive protein, leukocytes, creatinine, fluid balance, phosphate, sodium and chloride the maximal recorded value was used for model fitting. For the Horowitz index, the minimal recorded value per patient was used for model estimation. Results from the univariate analyses are shown in Additional file 1: Table S2

*AF* Atrial fibrillation, *AV* Atrio-ventricular, *CABG* Coronary artery bypass grafting

## Discussion

The main findings of our study are that new onset AF was significantly associated with an inflammatory response in multivariate analysis as indicated by increased CRP concentration, whereas fluid overload was significantly associated with postoperative AF only in univariate analyses. Reduced postoperative plasma phosphate concentration represented a unique risk factor for intermittent/permanent AF in a multiple logistic regression.

### Clinical outcomes

New onset and intermittent/permanent AF significantly impacted clinical outcomes of cardiac surgery patients. Patients of both AF groups had a median increase in ICU length of stay of approx. 2 days and required prolonged mechanical ventilation, which leads to increased hospital resource utilisation and higher costs for treatment [3]. Furthermore, new onset AF was significantly associated with an increased rate of dialysis (8.2% vs. 1.9% in the no AF group). In line with this finding, Tsai and co-workers and Aranki et al. previously described a significantly higher incidence of renal failure in subjects who developed AF after CABG [3, 10]. The hospital mortality rate was also significantly higher in patients with new onset AF compared to subjects without this arrhythmia. While Villareal and colleagues also identified postoperative AF as a significant risk factor for hospital mortality [11], more studies have reported lower long-term survival rates for patients who developed AF after surgery [10, 11, 14, 16, 18].

### Postoperative AF and inflammation

CRP concentration was the most significant predictor of new onset AF in multiple logistic regression analysis, pointing out a strong link between AF and inflammation. The effect was present even after accounting for the impact of age, which is reported to be the most powerful predictor of new onset AF [3–5, 7, 8, 11–14, 18–20]. Elevated levels of CRP as a rough marker for systemic inflammation have been reported in nonsurgical patients with atrial arrhythmias [22]. In line with our data, preoperative CRP concentration over 3.0 mg/dL significantly increased the risk for AF following CABG in a sample of 152 patients [23]. In supplement to these previous reports, we managed to demonstrate that higher postoperative CRP concentration as well is a significant predictor of new onset AF in our larger sample of 779 patients.

White blood cell count was significantly higher in patients with new onset AF compared to no AF patients on days 2 and 3 and in patients with intermittent/permanent AF relative to the disappeared AF group on days 4 and 5 (Fig. 3). These results are in line with the findings by Abdelhadi and associates, who reported elevated white blood cell count in patients with AF following CABG or valve surgery. The difference between the two groups was statistically significant on postoperative days 3 to 5 [24]. Notably, Abdelhidi et al. investigated the association between higher leukocyte concentration and new onset AF only. The new finding of our study is the observation that patients with intermittent/permanent AF also have significantly increased white blood cell count.

Patients with new onset AF had significantly more positive fluid balance on the day of surgery and postoperative days 1 and 2 (Fig. 2). This finding is in agreement with previous reports, which strongly implies fluid overload as a key player in the initiation of postoperative AF [8, 19, 25]. In a prospective observational cohort study in critically ill nonsurgical patients, Shaver and associates reported that new onset AF was associated with a more pronounced positive fluid balance as compared to fluid balance in patients with recurrent AF [26]. The authors postulated that new onset AF occurs in the context of prolonged hypotension and insufficient oxygen delivery and clinical management of these conditions may contribute to developing new onset AF. In contrast, a recurrent arrhythmic episode in patients with previous history is more likely the result of underlying structural changes in the atrial myocardium [26].

Interestingly, more positive fluid balance and leukocyte concentration were not significantly associated with new onset AF in the multivariable model despite significant differences in the univariate analyses, which might be due to other confounding factors.

Indeed, the connection between inflammation and AF indicated by CRP, positive fluid-balance, and leukocyte count is striking. The pathophysiology of AF is a multifactorial process and the exact role of inflammation has not been elucidated yet. However, it is generally believed that the inflammatory response promotes electrical and structural remodelling of the myocardium thereby increasing the risk for AF [27]. For example, membrane potential fluctuations might be a direct consequence of inflammation [28]. Furthermore, fibrosis or scarring of the myocardium might lead to heterogeneous electrical conduction which in turn might contribute to the emergence of ectopic beats, late potentials or wavelet re-entry [28]. In this context, the specific association between increased CRP levels and AF might be explained by the fact that CRP is just a marker for systemic inflammation. Another possibility is that CRP is involved in modulating the local inflammatory response by binding to phosphocholine and thus identifying phospholipid components of damaged cells and activating the complement system [29]. This molecular mechanism might suggest a more direct impact of CRP on the initiation of AF. Additionally, Watanabe and colleagues reported that CRP levels correlated with larger left atrial diameter in patients with paroxysmal AF [30], which is also a recognized risk factor for AF [4, 7]. Fluid overload might contribute to the development of postoperative arrhythmias in a similar manner, since Shaver and colleagues reported increased left atrial size in their study in critically ill patients with new onset AF who were also associated with more positive fluid balance [26]. However, it is difficult to distinguish between cause and consequence in our study. Positive fluid balance might have been the therapeutic attempt to tackle AF-induced hypotension and as such a consequence of AF. Another possibility is that inflammation might have led to positive fluid balance and AF is a cardiac facet of this pathophysiology without a causal link between both observations. Nevertheless, our findings give reason to prospective trials to reduce systemic inflammation with the aim of mitigating severe complications like AF which impair patient’s outcome.

### Potential risk factors for new onset and intermittent/permanent AF

Higher creatinine concentration was another significant predictor for new onset AF in multivariable regression analysis. This had already been suggested by Zaman and colleagues in a univariate analysis in CABG patients [12]. Conversely, higher Horowitz index values were associated with reduced occurrence of new onset AF. In this context, Haïssaguerre and colleagues reported in 45 patients that the majority of ectopic beats triggering AF originate in the pulmonary veins [31]. Impaired pulmonary function may thus induce ectopic beats and thereby promote the occurrence of AF [32]. Therefore, Horowitz index as marker for impaired pulmonary function might be a useful indicator for patients at high risk for developing new onset AF. Finally, a combined procedure of CABG and valve surgery significantly increased the risk for postoperative new onset AF compared to CABG alone. It is noteworthy that the more complex combined procedure is associated with longer CPB and ischemia time and thus an increased surgical injury, fostering inflammation following cardiac surgery [33, 34] Increased inflammatory response compared to an isolated CABG procedure might in turn lead to a higher incidence of postoperative new onset AF after a combined intervention.

In the multiple logistic regression analysis, higher creatinine and lower phosphate concentrations were significantly associated with postoperative intermittent/permanent AF (Table 4). Shwartz et al. conducted a study in this setting and reported a significant association between hypophosphatemia and increased incidence of newly occurring cardiac arrhythmias including AF in septic patients [35, 36]. In addition, Švagždienė and Širvinskas described significantly reduced postoperative phosphate concentrations in patients who developed new onset AF after CABG compared to patients who remained in sinus rhythm [37]. Interestingly, lower phosphate concentration only predicted intermittent/permanent AF but not new onset AF in our study. Notably, we managed to demonstrate this association in a multivariable regression analysis whereas previous reports on the link between hypophosphatemia and new onset AF were only derived from univariate analyses.

### Limitations of the analysis

The main limitation of our study is its retrospective nature. Therefore, unobserved confounding effects on the results we obtained cannot be excluded. This limitation is, however, offset by the explorative nature of the analysis. Furthermore, there were missing values in the data set regarding the daily fluid balance and clinical chemistry parameters. However, in the logistic regression analyses, this limitation was overcome by applying the multiple imputation methodology. Additionally, there might be more sophisticated parameters to describe inflammation with higher precision, e.g. interleukins or tumour necrosis factors. Since they were not part of routine laboratory testing, we could not include them in our analysis. Ultimately, we believe that investigating these variables would not have altered the message of this paper.

## Conclusions

In our retrospective analysis of patients undergoing elective cardiac surgery, we demonstrated that new onset AF and intermittent/permanent AF are clinically relevant postoperative complications associated with additional co-morbidities and increased ICU length of stay. In a multiple logistic regression analysis, new onset AF was significantly associated with increasing age, impaired renal and pulmonary function and systemic inflammation indicated by higher CRP concentrations. In contrast, lower phosphate concentrations occurred along with intermittent/permanent AF among patients with pre-existing AF. These findings suggest that personalized clinical management strategies for the prevention and treatment of both types of postoperative arrhythmias are warranted and interventions modulating (hyper)-inflammation might be promising.

## Additional files

Additional file 1: Supplementary material (Table S1: Univariate logistic regression analyses for new onset atrial fibrillation and Table S2: Univariate logistic regression analyses for intermittent/permanent atrial fibrillation). (DOCX 53 kb)

Additional file 2: Supplementary material (Figure S1: Plasma creatinine concentration changes over the time course of ICU stay, Figure S2: Horowitz index over the time course of ICU stay for new onse AF vs no AF patients and Figure S3: Plasma phosphate concentration changes over the time course of ICU stay for disappeared AF vs intermittent/permanent AF patients). (DOCX 391 kb)

## Abbreviations

AF: Atrial fibrillation
ALT: Alanine transaminase
AST: Aspartate transaminase
AV block III: Third degree (complete) atrio-ventricular block
CABG: Coronary artery bypass graft
CI: Confidence interval
CPB: Cardio-pulmonary bypass
CRP: C-reactive protein
ICU: Intensive care unit
IQR: Interquartile range
L: Liter
Ns: Not significant
OR: Odds ratio
Vs: Versus
