# Supplementary material for: Clinical significance and risk factors for new onset and recurring atrial fibrillation following cardiac surgery - a retrospective data analysis
Source: BMC Anesthesiol. 2017 Dec 2;17:163. doi: 10.1186/s12871-017-0455-7 (PMC5712135; doi:10.1186/s12871-017-0455-7)
Supplement: Supplementary file 1 — Supplementary material (Table S1: Univariate logistic regression analyses for new onset atrial fibrillation and Table S2: Univariate logistic regression analyses for intermittent/permanent atrial fibrillation). (DOCX 53 kb) [file 12871_2017_455_MOESM1_ESM.docx]

Table S1: Univariate logistic regression analyses for postoperative new onset atrial fibrillation

| **Variable** | **p-Value** |
| --- | --- |
| Age [decades] | <0.0001 |
| Weight | 0.552 |
| Gender | 0.614 |
| AV block III | 0.573 |
| Ventricular arrhythmias | 0.019 |
| Type surgery (relative to CABG) Valve CABG + valve | 0.863 0.0001 |
| Epinephrine administration | 0.013 |
| Dobutamine administration | 0.178 |
| Postoperative fluid balance* | 0.0002 |
| Postoperative leukocyte count* | 0.999 |
| Postoperative C-reactive protein concentration* | <0.0001 |
| Postoperative creatinine concentration* | <0.0001 |
| Postoperative total bilirubin concentration* | 0.648 |
| Postoperative ALT concentration* | 0.036 |
| Postoperative AST concentration* | 0.299 |
| Postoperative Horowitz index^+^ | <0.0001 |
| Postoperative phosphate concentration* | 0.524 |
| Postoperative bicarbonate concentration* | 0.697 |
| Postoperative sodium concentration* | 0.492 |
| Postoperative potassium concentration* | 0.528 |
| Postoperative chloride concentration* | 0.341 |
| Postoperative calcium concentration* | 0.1 |

Candidate variables with p<0.1 were entered into a multivariable logistic regression analysis.

* The maximal recorded value was used for model fitting.

+ The minimal recorded value was used for model fitting.

ALT - Alanine transaminase; AST - Aspartate transaminase; AV - Atrio-ventricular; CABG - Coronary artery bypasses grafting

Table S2: Univariate logistic regression analyses for postoperative intermittent/permanent atrial fibrillation

| **Variable** | **p-Value** |
| --- | --- |
| Age [decades] | 0.512 |
| Weight | 0.320 |
| Gender | 0.980 |
| AV block III | 0.083 |
| Ventricular arrhythmias | 0.987 |
| Type surgery (relative to CABG) Valve CABG + valve | 0.542 0.603 |
| Epinephrine administration | 0.066 |
| Dobutamine administration | 0.661 |
| Postoperative fluid balance* | 0.02 |
| Postoperative leukocyte count* | 0.037 |
| Postoperative C-reactive protein concentration* | 0.0004 |
| Postoperative creatinine concentration* | 0.008 |
| Postoperative total bilirubin concentration* | 0.185 |
| Postoperative ALT concentration* | 0.344 |
| Postoperative AST concentration* | 0.283 |
| Postoperative Horowitz index^+^ | 0.003 |
| Postoperative phosphate concentration* | 0.022 |
| Postoperative bicarbonate concentration* | 0.553 |
| Postoperative sodium concentration* | 0.005 |
| Postoperative potassium concentration* | 0.408 |
| Postoperative chloride concentration* | 0.012 |
| Postoperative calcium concentration* | 0.508 |

Candidate variables with p<0.1 were entered into a multivariable logistic regression analysis.

* The maximal recorded value was used for model fitting.

+ The minimal recorded value was used for model fitting.

ALT - Alanine transaminase; AST - Aspartate transaminase; AV - Atrio-ventricular; CABG - Coronary artery bypasses grafting
