# Supplementary material for: Clinical significance and risk factors for new onset and recurring atrial fibrillation following cardiac surgery - a retrospective data analysis
Source: BMC Anesthesiol. 2017 Dec 2;17:163. doi: 10.1186/s12871-017-0455-7 (PMC5712135; doi:10.1186/s12871-017-0455-7)
Supplement: Supplementary file 2 — Supplementary material (Figure S1: Plasma creatinine concentration changes over the time course of ICU stay, Figure S2: Horowitz index over the time course of ICU stay for new onse AF vs no AF patients and Figure S3: Plasma phosphate concentration changes over the time course of ICU stay for disappeared AF vs intermittent/permanent AF patients). (DOCX 391 kb) [file 12871_2017_455_MOESM2_ESM.docx]

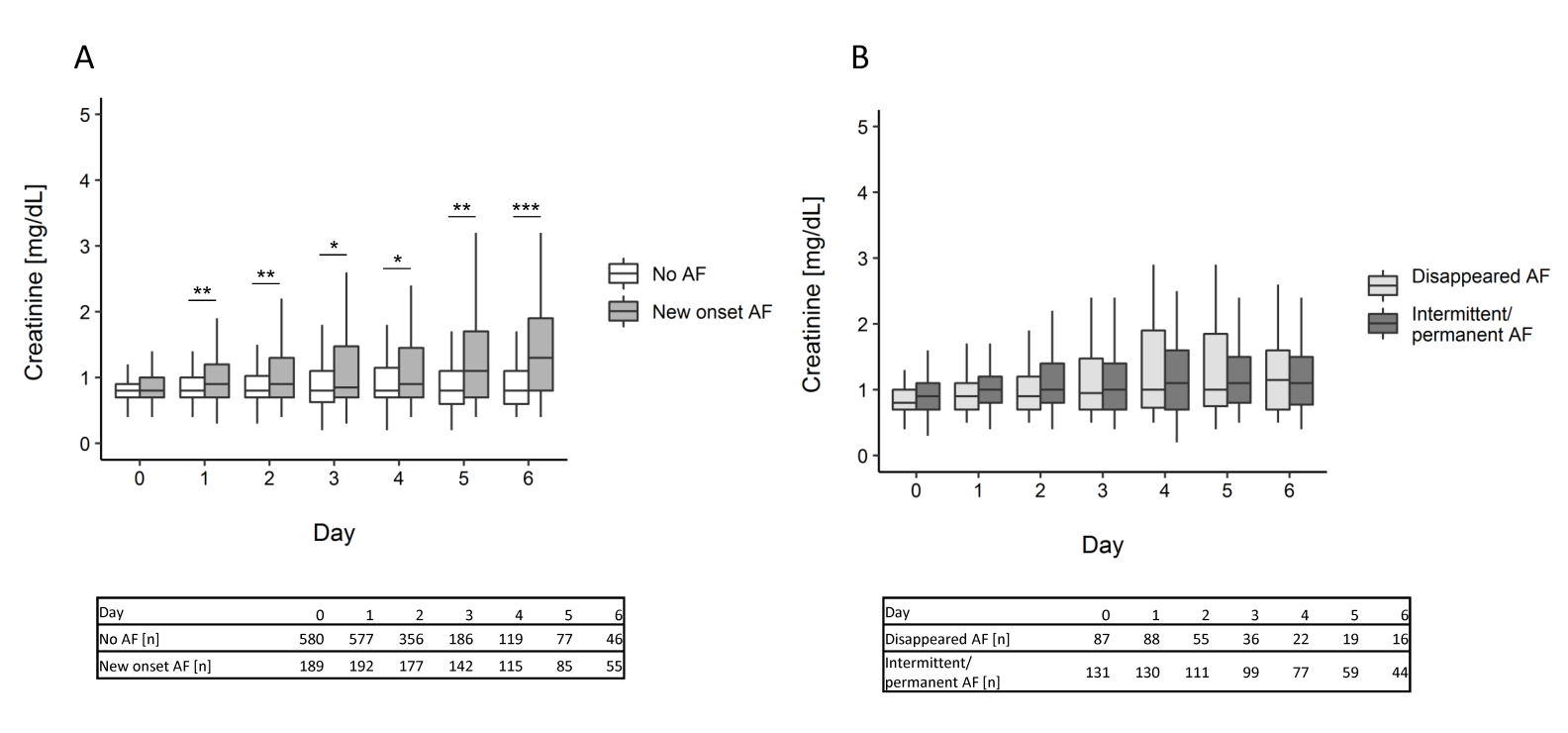


Figure S1: Plasma creatinine concentration changes over the time course of ICU stay

**A** Patients without atrial fibrillation (AF) versus patients with new onset AF. **B** Patients with disappeared AF versus patients with intermittent/permanent AF. Data are shown as median with 1^st^ and 3^rd^ quartile and minimum and maximum without outliers. Number of patients on each day is displayed under the graphs.

*** p<0.001, ** p<0.01, * p<0.05 (Wilcoxon-Mann-Whitney test)


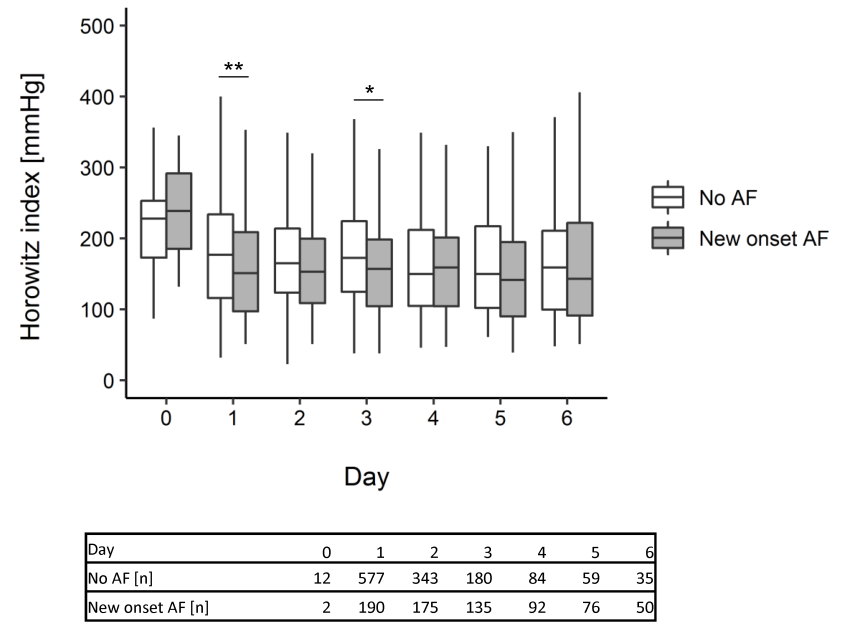


Figure S2: Horowitz index over the time course of ICU stay for new onse AF vs no AF patients

Data are shown as median with 1^st^ and 3^rd^ quartile and minimum and maximum without outliers. Number of patients on each day is displayed under the graphs.

** p<0.01, * p<0.05 (Wilcoxon-Mann-Whitney test)


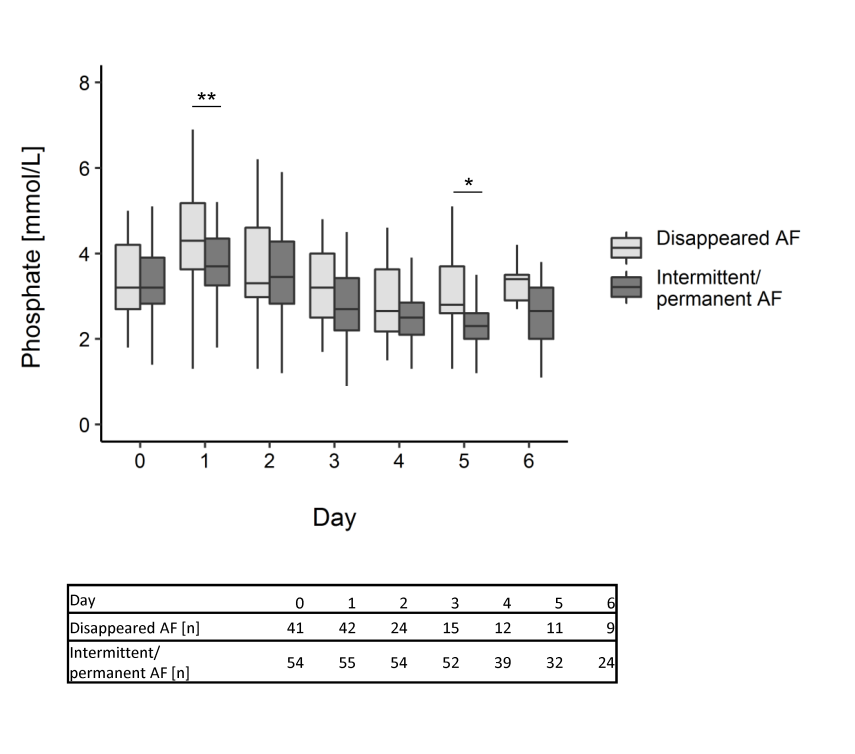


Figure S3: Plasma phosphate concentration changes over the time course of ICU stay for disappeared AF vs intermittent/permanent AF patients

Data are shown as median with 1^st^ and 3^rd^ quartile and minimum and maximum without outliers. Number of patients on each day is displayed under the graphs.

** p<0.01, * p<0.05 (Wilcoxon-Mann-Whitney test)
